# The Hypoxic Microenvironment of Breast Cancer Cells Promotes Resistance in Radiation Therapy

**DOI:** 10.3389/fonc.2020.629422

**Published:** 2021-02-18

**Authors:** Cordell Gilreath, Marjan Boerma, Zhiqiang Qin, M. Keith Hudson, Shanzhi Wang

**Affiliations:** ^1^ Chemistry Department, University of Arkansas at Little Rock, Little Rock, AR, United States; ^2^ Pharmaceutical Sciences, University of Arkansas for Medical Sciences, Little Rock, AR, United States; ^3^ Department of Pathology, University of Arkansas for Medical Sciences, Little Rock, AR, United States

**Keywords:** breast cancer, radiation therapy, hypoxia, free radicals, superoxide ions, radiation resistance

## Abstract

The American Cancer Society has estimated an expected 279,100 new breast cancer cases, and an expected 42,690 breast cancer deaths in the U.S. for the year 2020. This includes an estimated 276,480 women who are expected to be diagnosed. Radiation therapy, also called ionizing radiation therapy, is one of the most frequently used methods in the treatment of breast cancer. While radiation therapy is used in the treatment of more than 50% of all cancer cases, tumor resistance to ionizing radiation presents a major challenge for effective cancer treatment. Most tumor cells are in a hypoxic microenvironment that promotes resistance to radiation therapy. In addition to radiation resistance, the hypoxic microenvironment also promotes cancer proliferation and metastasis. In this review, we will discuss the hypoxic microenvironment of breast cancer tumors, related signaling pathways, breast cancer stem-like cells, and the resistance to radiation therapy. Recent developments in our understanding of tumor hypoxia and hypoxic pathways may assist us in developing new strategies to increase cancer control in radiation therapy.

## Introduction

Breast cancer is the second leading cause of cancer death in women. The American Cancer Society has estimated an expected 279,100 new breast cancer cases and an expected 42,690 breast cancer deaths in the U.S. for the year 2020 (1). There have been many advancements in the diagnosis and treatment of breast cancer in the past few decades. However, more research is still needed to overcome cancer resistance to therapy and improve the prognosis of advanced-stage breast cancer.

One significant obstacle to improve prognosis is breast cancer recurrence that is often associated with metastasis (2, 3). Breast cancer recurrence is the return of breast cancer months to years after the completion of initial treatment. Some cancer cells survive initial treatment and become undetected. These cancer cells may multiply and repopulate in nearby or distant areas. As such, the three types of breast cancer recurrence are local, regional, and metastatic recurrence. Local recurrence is the return of cancer in the same area of the breast as initial cancer; Regional recurrence is the return of cancer in the lymph nodes near the original cancer location; Metastatic recurrence, also called distant recurrence, is the return of breast cancer in areas distant from the original cancer site (1). Common metastatic sites include the bone, lungs, or brain, and these metastatic recurrences are the foremost cause of breast cancer death (4).

Radiation therapy is used as an adjunct therapy for many primary cancers and is one of the most frequently used methods in breast cancer therapy. Ionizing radiation targeted at breast cancer cells causes an interaction with water and O_2_ molecules near and inside the cells. This interaction produces free radicals and superoxide ions, which in turn cause damage to the cancer cell’s DNA and other macromolecules, and potentially induces cell death (5). The primary purposes of radiation therapy are to improve prognosis of primary treatments, to treat metastasized cancer cells, and to decrease the chance of recurrence. However, tumor resistance to ionizing radiation presents a major challenge for effective breast cancer treatment. Tumor resistance may be in part due to a hypoxic microenvironment that is common in tumors. Here, we outline the cellular response to ionizing radiation and signal transduction pathways induced by hypoxic conditions as targets to identify novel strategies to increase the efficacy of radiation therapy.

## Ionizing Radiation

**Figure 1:**
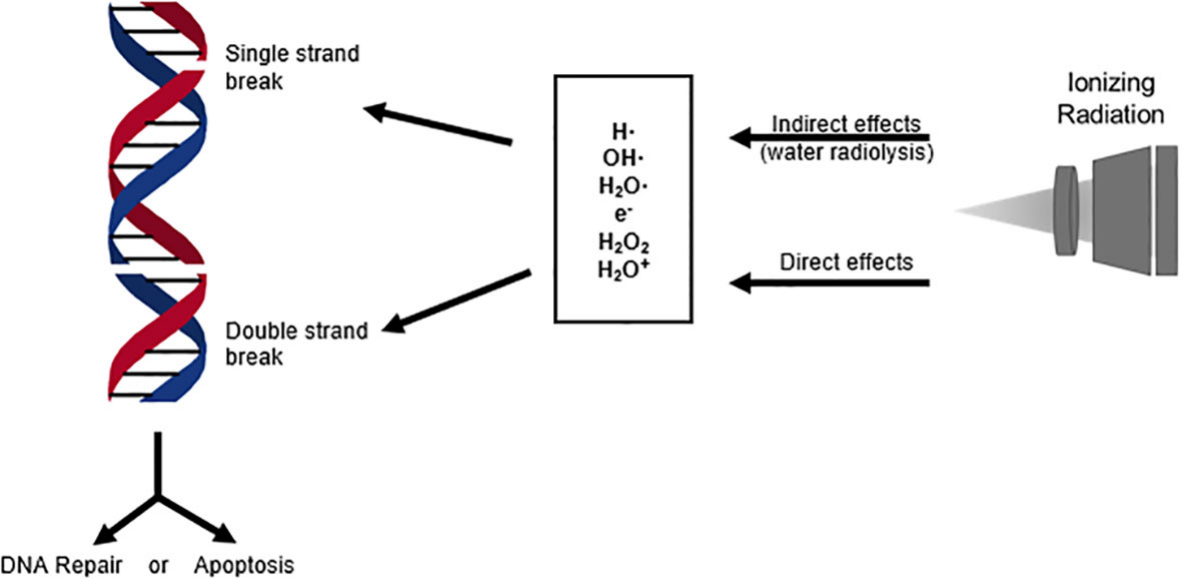
DNA breaks induced by direct and indirect effects of ionizing radiation activate cellular stress response mechanisms. These response mechanisms can either repair the DNA damage or signal the activation of apoptosis, which is the primary objective in cancer radiation therapy.

Ionizing radiation in breast cancer therapy is greatly dependent on the damaging effects of low linear energy transfer (Low LET) radiation, such as X rays (5). There are direct and indirect effects of ionizing radiation. Direct effects of ionizing radiation are direct interactions between the particle and the targeted macromolecule, such as DNA (**Figure 1**), which eventually can lead to cell death (5, 6). Indirect effects of ionization consist of an intermediate step between radiation and the macromolecules, such as in water radiolysis. During water radiolysis in radiation therapy, water molecules are decompositioned by ionization radiation, and several types of free radicals are generated to damage macromolecules. These free radicals primarily include the hydrated electron (e^-^_aq_), the hydrogen radical (H•), and the hydroxyl radical (OH•), which are highly reactive to the adjacent macromolecules (5). During radiaton therapy, the majority of deposited radiation will be absorbed by cellular water. This makes indirect ionization of water the primary cause of biological damage from radiation exposure (5).

## The Hypoxic Microenvironment

**Figure 2:**
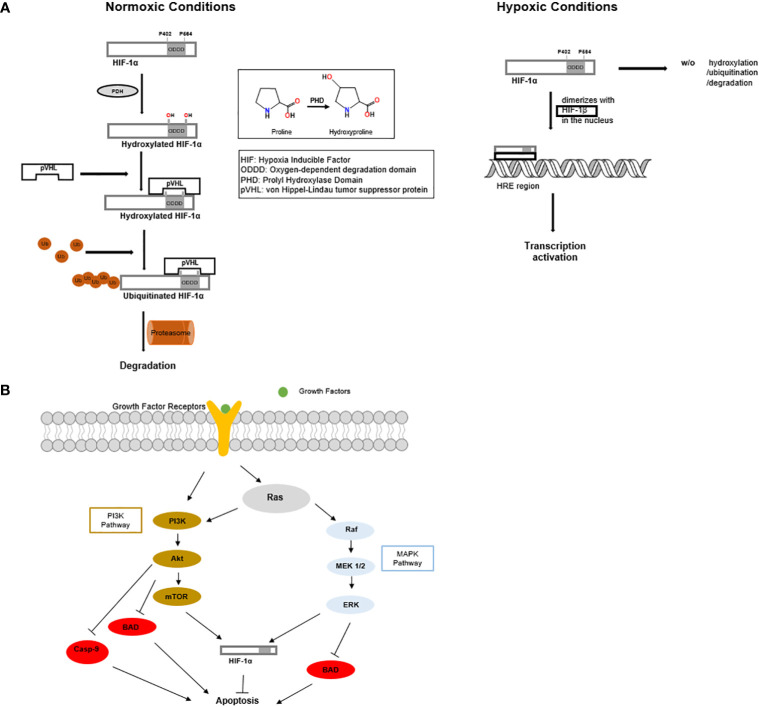
**(A)** Under normoxic conditions, HIF-1α is produced and rapidly undergoes hydroxylation, ubiquitination, and proteasomal degradation. While under hypoxic conditions, HIF-1α does not undergo proteasomal degradation. HIF-1α enters the nucleus and dimerizes with HIF-1β to form a stable heterodimer. The HIF-1 heterodimer promotes tumor growth and angiogenesis. **(B)** MAPK and PI3K signaling cascades inhibit apoptosis and promote the function of HIF1α.

Tumor hypoxia, which is the lack of oxygen within a tumor, is one of the most common characteristics of the tumor microenvironment due to rapid cell growth and oxygen consumption (7). The hypoxic microenvironment in breast cancer requires the tumor to adapt in order to survive, and as such, tumor hypoxia has been closely associated with angiogenesis, metastasis, chemoresistance, and radiation resistance (8–10). Hypoxia has been recognized to activate many signaling transduction pathways, such as RAS/RAF/and mitogen-activated protein kinase (MAPK) (11). Hypoxia within the tumor microenvironment activates the heterodimer hypoxia-inducible factor 1 (HIF-1), a transcription factor consisting of two protein subunits, HIF-1α and HIF-1β. The expression and function of HIF-1α is regulated by oxygen concentration, while HIF-1β is constitutively expressed. Under normoxic conditions, HIF-1α is hydroxylated at proline residues 402 and 564, then ubiquitinated by prolyl-hydroxylase domain enzymes (PHD), which leads to proteasomal degradation (**Figure 2A**) (12–14). Under hypoxic conditions, HIF-1α is stabilized by dimerizing with HIF-1β (**Figure 2A**). Upon hypoxia, the HIF-1 heterodimer binds to the hypoxia response elements of multiple genes, which activates their transcription (**Figure 2A**) (8, 15). Many of these gene products participate in metabolism, such as, glycolytic enzymes, glucose transporters, antigenic growth factors, and carbonic anhydrases. The upregulation of these genes in breast cancer mediate a metabolic change from oxidative to glycolytic (11, 16, 17). Intratumoral hypoxia and alterations of the tumor microenvironmentare mechanisms that increase HIF-1α levels in breast cancer. In addition, the mutation and inactivation of tumor suppressor genes such as the von Hippel-Lindau tumor suppressor (pVHL), tumor protein p53 (p53)), and phosphatase and tensin homolog (PTEN) are associated with increased HIF-1α activity (18). It is important to note that although HIF-1α is widely recognized as the main regulator in tumor hypoxia, many additional factors, such as histone acetyltransferase (p300) and the CREB-binding protein (CBP) (19, 20), are essential to promote the comprehensive hypoxic response within the tumor microenvironment (11).

## Hypoxic and Anti-Apoptotic Signaling Pathways

Hypoxic signaling within the tumor microenvironment is used by cancer cells to communicate with other cells and their extracellular environment. This communication within the hypoxic microenvironment is a highly explored area and is still not fully understood.

During hypoxia signaling, exosomes, the extracellular nanovesicles released by cells, play a vital role in communicating intercellular signals by way of paracrine signaling (7, 21). Tumor cells produce exosomes that contain several types of molecules from within the tumor cells, including miRNA, mRNA, DNA, proteins, and lipids that affect the activity of neighboring cells (21, 22). During hypoxia signaling, multiple pathways and even multiple cell types may crosstalk *via* exosomes. Boelens et al. showed that stromal cells released exosomes to communicate with breast cancer cells. This multi-signaling pathway uses both paracrine antiviral and juxtacrine neurogenic locus notch homolog protein 3 (NOTCH3) signaling to enhance breast cancer survival and therapy resistance. The communication is initiated by the stromal cells by increasing Ras-related protein Rab-27B (RAB27B) and transferring 5’-triphosphate RNA in exosomes. It results in the activation of retinoic acid-inducible gene 1 (RIG-l) antiviral signaling while simultaneously activates NOTCH3 receptors in breast cancer cells (23). The crosstalk between stromal and breast cancer cells signaling pathways converge as STAT1 promotes transcriptional responses to NOTCH3. This also promotes the initiation of producing tumor cell subpopulations that are prone to therapeutic resistance. These findings also suggest that blocking the NOTCH pathway resensitizes tumor cells to radiation and may be a therapeutic target in the treatment of cancer (24). The role of exosomes in the breast cancer tumor microenvironment is still not fully understood, but there is an established correlation between the activation of hypoxic signaling and increased exosome production (7). Research is currently underway to determine whether tumor cell exosomes may be a novel target in cancer therapy.

Several signaling pathways participate in breast cancer resistance to radiation therapy, such as Ras/phosphoinositide 3-kinase (PI3K)/PTEN/protein kinase B (Akt)/mammalian target of rapamycin (mTOR) (**Figure 2B**) (25), and Ras/Raf/mitogen-activated protein kinase kinase (MEK)/extracellular signal-regulated kinase (ERK) (MAPK) (**Figure 2B**) (26, 27).

The activation of Akt, as part of PI3K pathway, could directly promote therapeutic resistance, including resistance to radiation (26). Akt is a kinase that is activated through the phosphorylation of its two residues, threonine 308 and serine 473 (28, 29). As a promoter of cell division and growth, Akt also plays a role in response to DNA damage (28). Akt can deactivate the BCL2 family member BAD by phosphorylation (30) and deactivate the cysteine protease Caspase-9. The deactivation of BAD and Caspase 9 is detrimental to the promotion of apoptosis, which is an essential factor of therapeutic resistance in breast cancer treatment. PI3-K/Akt signaling pathway is dysregulated in breast cancer. Söderlund et al. showed that the stimulation of the epidermal growth factor erbB2 (HER2)/PI3-K/Akt with heregulin-B1 triggered the resistance to radiation-induced apoptosis in breast cancer (28). Furthermore, it was found that the inhibition of the PI3K signaling resulted in sensitizing brease cancer cell line BT-474, which overactivates PI3K pathway by overexpressing human epidermal growth factor receptor 2 (HER2). Independently, Steelman et al. showed that the PI3-k/PTEN/Akt/mTOR signaling cascade pathway was activated in breast cancer, therefore promoting its resistance to therapy. Consistently, the elevated levels of Akt-1 promoted resistance to doxorubicin, tamoxifen, and radiation. Interestingly, cells that were resistant to chemotherapy or radiation therapy harbored p53 mutations and expression of the downstream cyclin-dependent kinase inhibitor 1 (p21^Cip1^). Also, ERK, an enzyme associated with cell development and proliferation, was induced by Doxorubicin therapy (26). A better understanding of the mechanisms of these signaling cascades, the activation and inhibition of Akt, may present promising therapeutic targets in the treatment of breast cancer.

The MAPK signaling pathway is known to promote cancer cell survival and limit the effectiveness of radiation therapy (31). Criswell et al. showed that ionizing radiation activated the insulin-like growth factor -1 receptor (IGF-1R), which in turn activated the MAPK signaling pathway, which upregulates secretory clusterin (sCLU) expression, a stress induced pro-survival protein. The study presented evidence that AG1024, an IGF-1R inhibitor blocked the induction of sCLU after radiation (31). More research is necessary to fully understand the role of the delayed EGF-1R/MAPK signaling pathway, but inhibition of IGF-1R may be a potential target in cancer therapy.

The mammalian target of rapamycin (mTOR) is another signaling pathway closely related to radiation resistance in breast cancer. mTOR and ribosomal protein S6 Kinase Beta-1 (p-S6K1) were found to be elevated in breast cancer cells (32, 33). CD44^high^/CD24^low^ Michigan Cancer Foundation-7 (MCF-7) cells, a radioresistant breast cancer cell line, expresses higher levels of p-S6K1 than radiosensitive cells, suggesting a possible correlation between p-S6K1 and radiation resistance. Consistently, the inhibition of mTOR using everolimus increased radio-sensitivity in the CD44^high^/CD24^low^ MCF-7 cells (32). In radio-sensitivity prognosis, p-S6K1expression levels may be a predictor of therapeutic response and may also be a potential target to increase radiation therapy sensitivity (32).

Micro-RNA-21 (miR-21) suppresses the functions of many tumor suppressor genes, such as tropomyosin 1 (TPM1) and PTEN, which are associated with proliferation, apoptosis and metastasis (34–36). In the research study by Anastov et al., T47D, a radioresistant breast cancer cell line, and MDA-MB-361, a radiosensitive breast cancer cell line, were studied in parallel, miR-21 was found to be significantly elevated in the T47D cells, suggesting miR-21 contributes to radioresistance of breast cancers. The study presented evidence that miR-21 knockdown improved radiation induced apoptosis and growth arrest in radiation resistant cells comparable to that of radiation sensitive cells (35). It is well accepted that the overexpression of the anti-apoptotic miR-21 can stimulate cell cycle progression in the G2/M checkpoint. However, it is not established that the correlation between miR-21 and G2/M checkpoint arrest promotes radiation resistance. Nevertheless, the inhibition of miR-21 may be a potential therapeutic target and its overexpression a possible prognosis indicator (35).

Long non-coding RNA (lncRNA) HOX Transcript Antisense RNA (HOTAIR) has been shown in several studies to participate in the promotion and metastasis of breast cancer (37, 38), and its single nucleotide polymorphism is a marker for breast cancer. HOTAIR is upregulated in breast cancer, links DNA damage and nuclear factor kappa B (NF-κB) signaling and takes part in p53 regulated DNA damage response. The link between HOTAIR and the p53 and NF-κB pathways correlate with the promotion of breast cancer and radiation resistance. HOTAIR binds with many miRNAs in various cancer types, causing an upregulation of the miRNA targets and deviations in signaling transduction. Braunstein et al. showed that binding lncRNA HOTAIR with miR-218 resulted in a phenotypical radiation-sensitive breast tumor. The research suggested that the inhibition of HOTAIR could be a novel target in breast cancer treatment (39).

## Cancer Stem Cells

In recent decades, a tumor cell population with cancer stem cell (CSC) phenotype has accumulated attention for its role in resistance to treatments. These cells have the ability to self-renew and initiate subpopulations of differentiated progeny (40). Cancer stem cells have been identified in a variety of tumors, including brain cancers, breast cancers, prostate, and melanoma (41). This population of cancer cells has presented evidence of resistance to radiation therapy and chemotherapy (42–44). Research has presented evidence that the hypoxic tumor microenvironment is ideal for CSC survival (45). Further research is needed to better understand the role of the CSC phenotype subpopulation, but this subpopulation may be a new target to increase radiation sensitivity in cancer therapy.

Lin28 is a stem cell marker that is associated with radiation resistance in breast cancer (46). Apoptotic proteins poly(ADP-ribose) polymerase (PARP), caspase-3, and caspase-9 have significantly lower cleavage levels, thus less activation, in Lin28 overexpressing cells (46). As such, it was suggested that the overexpression of Lin28 mediated radioresistance by inhibiting radiation-induced apoptosis. Additionally, it has been shown that the Let-7 miRNA is downregulated in association with upregulation of Lin28 (47); and when the stabilized cells are transfected with Let-7 miRNA precursor, radiation sensitivity is resumed (46). Lin28 regulates Let-7 by directly interacting with the precesors of Let-7 family members (48). This suggests that Lin28 and Let-7 could be used as predictive biomarkers of response to radiation therapy.

The stem cell marker CD44^+^/CD24^-^ is recognized primarily in triple-negative breast cancer (TNBC) stem cells (49). CD44^+^/CD24^-^ has been associated with breast cancer resistance to ionizing radiation. In a 2011 study, Yin et al. showed that BRCA1 and Ataxia-Telangiectasia Mutated Kinase (ATM) activity are increased in CD44^+^/CD24^-^ cells (42). As an initiating factor for homologous recombination (HR), ATM is essential for the repair of radiation-induced double-strand DNA breaks (27, 50). ATM, a Ser/Thr kinase itself, is activated by autophosphorylation during double-strand DNA breaks. In CD44^+^/CD24^-^ cell lines and the primary culture of patient breast cancer cells, elevation in both expression and phosphorylation of ATM were found. Inhibition of ATM increased radiation sensitivity of the isolated CD44^+^/CD24^-^ cell, which suggests that ATM is a potential target to improve radiation sensitivity in breast cancer therapy (42).

Many studies have verified the breast cancer stem cell line with the CD44^+^/CD24^-^/ALDH^+^ marker, and recently the high expression of aldehyde dehydrogenase (ALDH^+^) was associated to therapeutic resistance. Croker et al. reported on the roles of ALDH^+^/CD44^+^ in breast cancer, where ALDH^+^/CD44^+^ was associated with chemoresistance, radiation resistance, poor prognosis, and played a role in metastasis (51). Considering that these stem cells are more resistant to therapy and promote proliferation in tumors (52), they may be more prone to distant metastasis. Additionally, it has been shown that this subset of cells expressed higher levels of therapy resistance proteins, p-glycoprotein, GSTpi, and/or CHK1 (51). Consistently, inhibition of ALDH^+^ using diethylaminobenzaldehyde (DEAB) or all trans retinoic acid (ATRA) resulted in significantly improved radiation sensitivity, suggesting ALDH could be a potential target for improving therapeutic results (51).

## Conclusion

As one of the most used methods to treat breast cancer, ionizing radiation in radiation therapy creates reactive oxygen species that cause cell damage and induce cell death. The hypoxic microenvironment of breast cancer cells promotes tumor cell proliferation, apoptosis resistance, metastasis, and resistance to radiation as well as other therapeutics. The overexpression and stabilization of the protein HIF-1α do not only result from low oxygen levels within the microenvironment, but also promote the advancement of hypoxia and facilitates tumor cell survival within the hypoxic microenvironment. The cancer cell’s capacity to survive in a low oxygen environment presents a major challenge to effective radiation therapy. In addition, the adaptation to the hypoxic microenvironment also promotes additional alterations, including metabolic changes, mutations, signaling pathways, upregulation and downregulation of various cellular components. Many of these adaptations decrease radiation sensitivity.

Extensive studies have been performed to elucidate the hypoxic response mechanisms, anti-apoptotic pathways, and cascades that lead to resistance to radiation. This led to the discovery of promising therapeutic targets for drug development to sensitize tumors to radiations. Importantly, the presence of the radiosensitizing targets will be critical to predict the prognosis after radiaotherpy. To achieve these goals, a deeper understanding of the development of radiation resistance in breast cancers, especially for the subgroups, is needed to develop specified and personalized therapy.

## Author Contributions

All authors listed have made a substantial, direct, and intellectual contribution to the work and approved it for publication.

## Funding

This publication was made possible by the Arkansas INBRE program, supported by a grant from the National Institute of General Medical Sciences, (NIGMS), P20 GM103429 from the National Institutes of Health.

## Conflict of Interest

The authors declare that the research was conducted in the absence of any commercial or financial relationships that could be construed as a potential conflict of interest.

## References

[1] Siegel RL Miller KD Jemal A. Cancer statistics, 2020. CA Cancer J Clin (2020) 70(1):7–30. 10.3322/caac.21590 31912902

[2] Hagemeister FB Buzdar AU Luna MA Blumenschein GR. Causes of death in breast cancer a clinicopathologic study. Cancer (1980) 46(1):162–7. 10.1002/1097-0142(19800701)46:1<162::AID-CNCR2820460127>3.0.CO;2-B 7388758

[3] Cristofanilli M Hayes DF Budd GT Ellis MJ Stopeck A Reuben JM. Circulating tumor cells: A novel prognostic factor for newly diagnosed metastatic breast cancer. J Clin Oncol (2005) 23(7):1420–30. 10.1200/JCO.2005.08.140 15735118

[4] Hara T Iwadate M Tachibana K Waguri S Takenoshita S Hamada N. Metastasis of breast cancer cells to the bone, lung, and lymph nodes promotes resistance to ionizing radiation Takamitsu. Strahlentherapie Und Onkol (2017) 193(10):848–55. 10.1007/s00066-017-1165-2 28642964

[5] Dance DR Christofides S Maidment M Mclean I Ng KH. Diagnostic Radiology Physics: A handbook for teachers and students. Iaea (2014) 710.

[6] Truong T Sun G Doorly M Wang JYJ Schwartz MA. Modulation of DNA damage-induced apoptosis by cell adhesion is independently mediated by p53 and c-Abl. Proc Natl Acad Sci U S A (2003) 100(18):10281–6. 10.1073/pnas.1635435100 PMC193552 12928501

[7] King HW Michael MZ Gleadle JM. Hypoxic enhancement of exosome release by breast cancer cells. BMC Cancer (2012) 12:421. 10.1186/1471-2407-12-421 22998595 PMC3488584

[8] Krishnamachary B Penet MF Nimmagadda S Mironchik Y Raman V Solaiyappan M. Hypoxia Regulates CD44 and Its Variant Isoforms through HIF-1α in Triple Negative Breast Cancer. PloS One (2012) 7(8):1–9. 10.1371/journal.pone.0044078 PMC3429433 22937154

[9] Park JE Tan HS Datta A Lai RC Zhang H Meng W. Hypoxic tumor cell modulates its microenvironment to enhance angiogenic and metastatic potential by secretion of proteins and exosomes. Mol Cell Proteomics (2010) 9(6):1085–99. 10.1074/mcp.M900381-MCP200 PMC2877972 20124223

[10] Dales JP Garcia S Meunier-Carpentier S Andrac-Meyer L Haddad O Lavaut MN. Overexpression of hypoxia-inducible factor HIF-1α predicts early relapse in breast cancer: Retrospective study in a series of 745 patients. Int J Cancer (2005) 116(5):734–9. 10.1002/ijc.20984 15849727

[11] Brennan DJ Jirstrom K Kronblad Å Millikan RC Landberg G Duffy MJ. CA IX is an independent prognostic marker in premenopausal breast cancer patients with one to three positive lymph nodes and a putative marker of radiation resistance. Clin Cancer Res (2006) 12(21):6421–31. 10.1158/1078-0432.CCR-06-0480 17085655

[12] Jokilehto T Rantanen K Luukkaa M Heikkinen P Grenman R Minn H. Overexpression and nuclear translocation of hypoxia-inducible factor prolyl hydroxylase PHD2 in head and neck squamous cell carcinoma is associated with tumor aggressiveness. Clin Cancer Res (2006) 12(4):1080–7. 10.1158/1078-0432.CCR-05-2022 16489060

[13] Lee G Won HS Lee YM Choi JW Oh TI Jang JH. Oxidative Dimerization of PHD2 is Responsible for its Inactivation and Contributes to Metabolic Reprogramming via HIF-1α Activation. Sci Rep (2016) 6(November 2015):1–12. 10.1038/srep18928 26740011 PMC4703963

[14] Zheng X Linke S Dias JM Zheng X Gradin K Wallis TP. Interaction with factor inhibiting HIF-1 defines an additional mode of cross-coupling between the Notch and hypoxia signaling pathways. Proc Natl Acad Sci USA (2008) 105(9):3368–73. 10.1073/pnas.0711591105 PMC2265116 18299578

[15] Blancher C Moore JW Talks KL Houlbrook S Harris AL. Relationship of hypoxia-inducible factor (HIF)-1α and HIF-2α expression to vascular endothelial growth factor induction and hypoxia survival in human breast cancer cell lines. Cancer Res (2000) 60(24):7106–13. 11156418

[16] Kim JW Tchernyshyov I Semenza GL Dang CV. HIF-1-mediated expression of pyruvate dehydrogenase kinase: A metabolic switch required for cellular adaptation to hypoxia. Cell Metab (2006) 3(3):177–85. 10.1016/j.cmet.2006.02.002 16517405

[17] Jiang BH Agani F Passaniti A Semenza GL. V-SRC induces expression of hypoxia-inducible factor 1 (HIF-1) and transcription of genes encoding vascular endothelial growth factor and enolase 1: Involvement of HIF-1 in tumor progression. Cancer Res (1997) 57(23):5328–35. 9393757

[18] Semenza GL. Regulation of metabolism by hypoxia-inducible factor 1. Cold Spring Harb Symp Quant Biol (2011) 76:347–53. 10.1101/sqb.2011.76.010678 21785006

[19] Arany Z Huang LE Eckner R Bhattacharya S Jiang C Goldberg MA. An essential role for p300/CBP in the cellular response to hypoxia. Proc Natl Acad Sci USA (1996) 93(23):12969–73. 10.1073/pnas.93.23.12969 PMC24030 8917528

[20] Xenaki G Ontikatze T Rajendran R Stratford IJ Dive C Krstic-Demonacos M. PCAF is an HIF-1α cofactor that regulates p53 transcriptional activity in hypoxia. Oncogene (2008) 27(44):5785–96. 10.1038/onc.2008.192 PMC2664615 18574470

[21] Zhang X Sai B Wang F Wang L Wang Y Zheng L. Hypoxic BMSC-derived exosomal miRNAs promote metastasis of lung cancer cells via STAT3-induced EMT. Mol Cancer (2019) 18(1):1–15. 10.1186/s12943-019-0959-5 30866952 PMC6417285

[22] Jabbari N Nawaz M Rezaie J. Ionizing radiation increases the activity of exosomal secretory pathway in MCF-7 human breast cancer cells: A possible way to communicate resistance against radiotherapy. Int J Mol Sci (2019) 20(15):3649. 10.3390/ijms20153649 PMC6696324 31349735

[23] Ruivo CF Melo SA. The emerging role of exosomes in cancer progression and their potential as therapy targets. In: Recent Trends in Cancer Biology: Spotlight on Signaling Cascades and MicroRNAs: Cell Signaling Pathways and MicroRNAs in Cancer Biology. (2018) 27–45. 10.1007/978-3-319-71553-7_3

[24] Boelens MC Wu TJ Nabet BY Xu B Qiu Y Yoon T. Exosome transfer from stromal to breast cancer cells regulates therapy resistance pathways. Cell (2014) 159(3):499–513. 10.1016/j.cell.2014.09.051 25417103 PMC4283810

[25] Kim KW Myers CJ Jung DK Lu B. NVP-BEZ-235 enhances radiosensitization via blockade of the PI3k/mTOR pathway in cisplatin-resistant non-small cell lung carcinoma. Genes Cancer (2014) 5(7–8):293–302. 10.18632/genesandcancer.27 25221647 PMC4162139

[26] Steelman LS Navolanic P Chappell WH Abrams SL Wong EWT Martelli AM. Involvement of Akt and mTOR in chemotherapeutic and hormonal-based drug resistance and response to radiation in breast cancer cells. Cell Cycle (2011) 10(17):3003–15. 10.4161/cc.10.17.17119 PMC3218601 21869603

[27] Ahmed KM Dong S Fan M Li JJ. Nuclear factor-κB p65 inhibits mitogen-activated protein kinase signaling pathway in radioresistant breast cancer cells. Mol Cancer Res (2006) 4(12):945–55. 10.1158/1541-7786.MCR-06-0291 17189385

[28] Söderlund K Pérez-Tenorio G Stål O. Activation of the phosphatidylinositol 3-kinase/Akt pathway prevents radiation-induced apoptosis in breast cancer cells. Int J Oncol (2005) 26(1):25–32. 10.3892/ijo.26.1.25 15586221

[29] Kirkegaard T Witton CJ McGlynn LM Tovey SM Dunne B Lyon A. AKT activation predicts outcome in breast cancer patients treated with tamoxifen. J Pathol (2005) 207(2):139–46. 10.1002/path.1829 16088978

[30] Widmann C Gibson S Johnson GL. Caspase-dependent cleavage of signaling proteins during apoptosis. A turn-off mechanism for anti-apoptotic signals. J Biol Chem (1998) 273(12):7141–7. 10.1074/jbc.273.12.7141 9507028

[31] Criswell T Beman M Araki S Leskov K Cataldo E Mayo LD. Delayed activation of insulin-like growth factor-1 receptor/Src/ MAPK/Egr-1 signaling regulates clusterin expression, a pro-survival factor. J Biol Chem (2005) 280(14):14212–21. 10.1074/jbc.M412569200 15689620

[32] Choi J Yoon YN Kim N Park CS Seol H Park IC. Predicting Radiation Resistance in Breast Cancer with Expression Status of Phosphorylated S6K1. Sci Rep (2020) 10(1)1–8. 10.1038/s41598-020-57496-8 31959810 PMC6971275

[33] Lin HJ Hsieh FC Song H Lin J. Elevated phosphorylation and activation of PDK-1/AKT pathway in human breast cancer. Br J Cancer (2005) 93(12):1372–81. 10.1038/sj.bjc.6602862 PMC2361529 16288304

[34] Iorio MV Ferracin M Liu CG Veronese A Spizzo R Sabbioni S. MicroRNA gene expression deregulation in human breast cancer. Cancer Res (2005) 65(16):7065–70. 10.1158/0008-5472.CAN-05-1783 16103053

[35] Anastasov N Höfig I Vasconcellos IG Rappl K Braselmann H Ludyga N. Radiation resistance due to high expression of miR-21 and G2/M checkpoint arrest in breast cancer cells. Radiat Oncol (2012) 7(1):1–12. 10.1186/1748-717X-7-206 23216894 PMC3573984

[36] Ma X Choudhury SN Hua X Dai Z Li Y. Interaction of the oncogenic miR-21 microRNA and the p53 tumor suppressor pathway. Carcinogenesis (2013) 34(6):1216–23. 10.1093/carcin/bgt044 PMC3670255 23385064

[37] Sørensen KP Thomassen M Tan Q Bak M Cold S Burton M. Long non-coding RNA HOTAIR is an independent prognostic marker of metastasis in estrogen receptor-positive primary breast cancer. Breast Cancer Res Treat (2013) 142(3):529–36. 10.1007/s10549-013-2776-7 24258260

[38] Özeş AR Wang Y Zong X Fang F Pilrose J Nephew KP. Therapeutic targeting using tumor specific peptides inhibits long non-coding RNA HOTAIR activity in ovarian and breast cancer. Sci Rep (2017) 7(1):1–11. 10.1038/s41598-017-00966-3 28420874 PMC5429858

[39] Hu X Ding D Zhang J Cui J. Knockdown of lncRNA HOTAIR sensitizes breast cancer cells to ionizing radiation through activating miR-218. Biosci Rep (2019) 29(4):1–9. 10.1042/BSR20181038 PMC6449517 30429228

[40] Lagadec C Vlashi E Della Donna L Meng YH Dekmezian C Kim K. Survival and self-renewing capacity of breast cancer initiating cells during fractionated radiation treatment. Breast Cancer Res (2010) 12(1):1–13. 10.1186/bcr2479 PMC2880434 20158881

[41] Phillips TM McBride WH Pajonk F. The response of CD24-/low/CD44+ breast cancer-initiating cells to radiation. J Natl Cancer Inst (2006) 98(24):1777–85. 10.1093/jnci/djj495 17179479

[42] Yin H Glass J. The phenotypic radiation resistance of CD44 +/CD24 -or low breast cancer cells is mediated through the enhanced activation of ATM signaling. PloS One (2011) 6(9):1–11. 10.1371/journal.pone.0024080 PMC3174160 21935375

[43] Griñán-Lisón C Olivares-Urbano MA Jiménez G López-Ruiz E del Val C Morata-Tarifa C. miRNAs as radio-response biomarkers for breast cancer stem cells. Mol Oncol (2020) 14(3):556–70. 10.1002/1878-0261.12635 PMC7053246 31930680

[44] Qi XS Pajonk F McCloskey S Low DA Kupelian P Steinberg M. Radioresistance of the breast tumor is highly correlated to its level of cancer stem cell and its clinical implication for breast irradiation. Radiother Oncol (2017) 124(3):455–61. 10.1016/j.radonc.2017.08.019 PMC6128144 28923575

[45] Lock FE McDonald PC Lou Y Serrano I Chafe SC Ostlund C. Targeting carbonic anhydrase IX depletes breast cancer stem cells within the hypoxic niche. Oncogene (2013) 32(44):5210–9. 10.1038/onc.2012.550 23208505

[46] Wang L Yuan C Lv K Xie S Fu P Liu X. Lin28 Mediates Radiation Resistance of Breast Cancer Cells via Regulation of Caspase, H2A.X and Let-7 Signaling. PloS One (2013) 8(6):6–11. 10.1371/journal.pone.0067373 PMC3688678 23840685

[47] Wang L Wang Y-X Zhang D-Z Fang X-J Sun P-S Xue H-C. Let-7a mimic attenuates CCL18 induced breast cancer cell metastasis through Lin 28 pathway. BioMed Pharmacother (2016) 78:301–7. 10.1016/j.biopha.2016.01.028 26898455

[48] Nam Y Chen C Gregory RI Chou JJ Sliz P. Molecular basis for interaction of let-7 MicroRNAs with Lin28. Cell (2011) 147(5):1080–91. 10.1016/j.cell.2011.10.020 PMC3277843 22078496

[49] Ma F Li H Wang H Shi X Fan Y Ding X. Enriched CD44+/CD24- population drives the aggressive phenotypes presented in triple-negative breast cancer (TNBC). Cancer Lett (2014) 353(2):153–9. 10.1016/j.canlet.2014.06.022 25130168

[50] Burma S Chen BP Murphy M Kurimasa A Chen DJ. ATM Phosphorylates Histone H2AX in Response to DNA Double-strand Breaks. J Biol Chem (2001) 276(45):42462–7. 10.1074/jbc.C100466200 11571274

[51] Croker AK Allan AL. Inhibition of aldehyde dehydrogenase (ALDH) activity reduces chemotherapy and radiation resistance of stem-like ALDH hiCD44 + human breast cancer cells. Breast Cancer Res Treat (2012) 133(1):75–87. 10.1007/s10549-011-1692-y 21818590

[52] Qiu Y Pu T Guo P Wei B Zhang Z Zhang H. ALDH+/CD44+ cells in breast cancer are associated with worse prognosis and poor clinical outcome. Exp Mol Pathol (2016) 100(1):145–50. 10.1016/j.yexmp.2015.11.032 26687806

